# Correlation of Sagittal Spinopelvic Alignment Parameters With Pain and Functional Disability in Chronic Non-specific Low Back Pain

**DOI:** 10.7759/cureus.95747

**Published:** 2025-10-30

**Authors:** Rajib Sarkar, Samriddhi Sarkar, Siddharth Keswani, Abhishek K Sah, Subhadeep Bhattacharjee

**Affiliations:** 1 Orthopaedics, ICARE Institute of Medical Science and Research, Haldia, IND; 2 Orthopaedics, Indira Gandhi Government General Hospital and Post Graduate Institute, Puducherry, IND

**Keywords:** chronic non- specific low back pain, lumbar lordosis, oswestry disability index, pelvic incidence, pelvic tilt, pi-ll mismatch, sacral slope, spinopelvic alignment, visual analogue scale (vas)

## Abstract

Introduction

This cross-sectional study evaluated the correlation between sagittal spinopelvic alignment parameters and pain and disability in patients with chronic non-specific low back pain (CNSLBP). While spinopelvic parameters like pelvic incidence (PI), pelvic tilt (PT), sacral slope (SS), and lumbar lordosis (LL) are key to sagittal alignment, their biomechanical relationship with functional impairment and pain in CNSLBP has not been fully quantified.

Methods

Ninety adults aged 21-60 years with CNSLBP were clinically and radiographically assessed. The Visual Analogue Scale (VAS) and Oswestry Disability Index (ODI) were used for clinical evaluation. Radiographic parameters (PI, PT, SS, LL, and Roussouly Type 1-4) were measured from lateral standing radiographs. Correlation and regression analyses were used to examine the relationships between these parameters and clinical scores.

Results

PI-LL mismatch showed a significant correlation with ODI (r = 0.29, p = 0.008) and a moderate association with VAS (r = 0.22, p = 0.06). Linear regression confirmed PI-LL mismatch as an independent predictor of disability (β = 0.251, p = 0.003). VAS was also positively correlated with ODI (r = 0.46, p < 0.001). Roussouly types were associated with structural parameters but not with clinical severity.

Conclusion

PI-LL mismatch is an important determinant of pain and disability in CNSLBP. The findings suggest that the impact of mismatch should be considered in accordance with an individual's pelvic incidence rather than a single fixed threshold. Future studies should investigate whether correcting the mismatch toward patient-specific targets improves clinical outcomes.

## Introduction

Chronic non-specific low back pain (CNSLBP), defined as low back pain lasting three months or more without a specific identifiable pathological cause, represents a major global health burden [1]. It is a leading cause of disability, significantly impacting quality of life, work productivity, and healthcare expenditure worldwide [2,3]. Despite its high prevalence and socioeconomic impact, the precise aetiology of CNSLBP remains elusive, making effective diagnosis and targeted treatment challenging [4]. While various factors like age-related degenerative changes, lifestyle, psychological stressors, and occupational demands are implicated, a clear understanding of biomechanical contributions is still emerging [5].

Recent research has increasingly focused on the significance of spinopelvic parameters, measurable radiographic angles, and distances that describe the alignment and interaction of the spine and pelvis, in maintaining sagittal balance and their potential role in the pathophysiology of low back pain [6,7]. Parameters such as pelvic incidence (PI), pelvic tilt (PT), sacral slope (SS), and lumbar lordosis (LL) are crucial for understanding the complex interplay between spinal curves and pelvic orientation [8]. A commonly employed classification system for LL, the Roussouly classification, categorizes spinal shapes based on the SS and the apex of LL [9], and has been associated with variations in spinal biomechanics and susceptibility to low back pain [8,10]. While the Roussouly classification provides a framework for understanding different LL types, its specific correlation with pain and functional disability in CNSLBP remains an area of ongoing investigation. Discrepancies or unfavourable relationships among these parameters can alter spinal biomechanics, potentially leading to increased mechanical stress on spinal structures, muscle imbalances, and ultimately, pain and functional limitations [11].

However, the direct correlation between these quantifiable spino-pelvic parameters and the subjective experience of pain and objective measures of functional disability in individuals with CNSLBP is not yet fully established. Existing literature often highlights the importance of sagittal alignment in specific spinal deformities or post-surgical outcomes, but a comprehensive understanding of their influence in the non-specific chronic pain population is less clear [7,12]. Therefore, this study aims to investigate the correlation between various spinopelvic parameters and the severity of pain and functional disability in patients suffering from CNSLBP, thereby contributing to a better understanding of the biomechanical factors associated with this debilitating condition.

## Materials and methods

Study design

This was a cross-sectional, observational study conducted at a tertiary care teaching hospital, ICARE Institute of Medical Sciences and Research & Dr. Bidhan Chandra Roy Hospital, Haldia, West Bengal, India, from January 2024 to August 2025. The study was approved by the Institutional Ethics Committee of ICARE Institute of Medical Sciences and Research (approval number: IIMSAR-Haldia/IEC/August 2025/25) and was conducted in accordance with the Declaration of Helsinki. CNSLBP was defined as low back pain localized between the costal margins and the inferior gluteal folds, persisting for more than three months, and not attributable to any specific pathological cause.

Study participants

A total of 90 consecutive adult patients aged between 21 and 60 years, presenting with CNSLBP and fulfilling the inclusion and exclusion criteria, were enrolled.

To standardize the sagittal spinal alignment, only participants with a wall-occiput distance (WOD) of 0 cm were included. Each participant was required to stand comfortably with the occiput, both shoulders, buttocks, and heels touching a vertical wall with knees extended. This criterion ensured the inclusion of participants with clinically global sagittal balance, and those with severe fixed sagittal plane deformities were excluded. Participants with a specific cause of chronic low back pain (e.g., spinal infection, tumour, fracture, inflammatory spondyloarthropathies, spondylolisthesis, coronal plane spinal curvature, cauda equina syndrome, with leg pain or claudication) were excluded from the study. Patients with systemic inflammatory diseases or autoimmune disorders and prior spinal surgery, and pregnant patients, were excluded from the study. Patients with conditions significantly affecting gait or posture (e.g., severe hip or knee osteoarthritis, neurological disorders affecting balance) were excluded from the study. Patients with an inability to understand or comply with instructions were also excluded from the study.

Sample size calculation

Based on a hypothesized moderate correlation (r = 0.3) between spinopelvic parameters and functional outcome scores, with α = 0.05 and power = 80%, the minimum required sample size was estimated to be 85 participants. To account for potential dropouts and data exclusions, a final target of 90 participants was set and successfully achieved.

Clinical assessment 

For each participant, a detailed clinical history was obtained, including pain duration, characteristics, and aggravating and alleviating factors. The physical examination included a thorough musculoskeletal and neurological assessment, and an evaluation of the lumbar spine's range of motion. These findings were correlated with radiological examinations, including an MRI, to rule out any underlying etiopathological causes. Each participant was examined by two orthopaedic spinal surgeons independently before inclusion as a CNSLBP case.

Pain and functional disability assessment

Pain intensity was quantified using the Visual Analogue Scale (VAS), ranging from 0 to 10, where 0 indicated no pain and 10 represented the worst imaginable pain [13].

Functional disability was assessed using the Oswestry Disability Index (ODI), a validated 10-item questionnaire, scored from 0% (no disability) to 100% (maximum disability) [14]. Permission for the use of the ODI version 2.1b was taken from Mapi Research Trust, Lyon, France (https://eprovide.mapi-trust.org).

Radiological assessment of spinopelvic parameters

All participants underwent standardized standing left lateral radiographs of the lumbosacral spine, including both femoral heads. Radiographs were obtained in a relaxed standing posture, with knees extended, both feet parallel, arms supported to avoid flexion, and ensuring WOD = 0.

**Figure 1:**
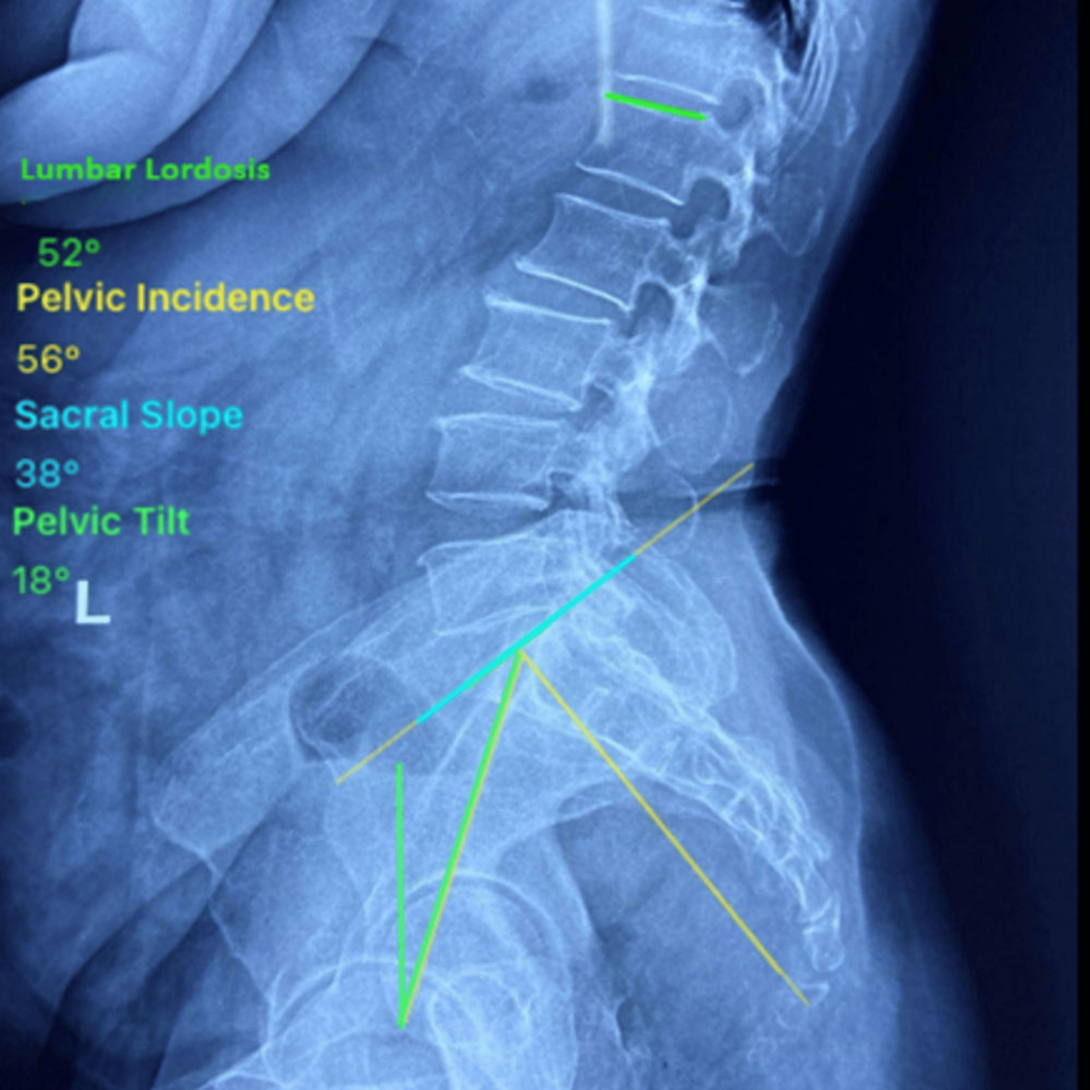
Measurement of spinopelvic parameters using Surgimap software on a plain standing lumbosacral radiograph The parameters include pelvic incidence (PI), pelvic tilt (PT), sacral slope (SS), and lumbar lordosis (LL)

Anteroposterior (AP) radiographs were also obtained to exclude coronal plane deformity. PI, PT, SS, LL (measured as Cobb angle between L1 superior endplate and S1 superior endplate), and Roussouly Classification of LL (Types 1 to 4) were measured using the Surgimap® software (Nemaris Inc., New York, United States) (Figure 1). All measurements were made independently by two trained observers who were blinded to the clinical data. Each parameter was recorded to the nearest degree, and the mean of two readings was considered for analysis.

Statistical analysis

Data were analyzed using IBM SPSS Statistics for Windows, version 26.0 (IBM Corp., Armonk, New York, United States). Descriptive statistics were presented as means ± standard deviations (SD) for continuous variables and frequencies with percentages for categorical variables. Correlations between spinopelvic alignment parameters and VAS/ODI scores were assessed using Pearson’s correlation for normally distributed variables and Spearman’s rank correlation for non-parametric variables. The association between Roussouly types and VAS/ODI scores was evaluated using one-way ANOVA (for normally distributed data) or Kruskal-Wallis test (for skewed data). Post hoc comparisons were performed where appropriate. A p-value < 0.05 was considered statistically significant.

## Results

The present study analyzed sagittal spinopelvic alignment parameters in patients with NSCNBP and their relationship with pain and disability. The mean age of participants was 46.2 years (SD = 10.6, range 21-60), with 48 female (53.3%) and 42 male (46.7%) participants. Pain scores were high, with a mean VAS of 6.9 (SD = 2.2), while the ODI averaged 20.5 (SD = 6.1), corresponding to an average disability of 41%, which falls within the moderate to severe disability range (Table 1).

**Table 1:** Descriptive statistics and normality tests of demographic and spinopelvic parameters SS, sacral slope; PI, pelvic incidence; PT, pelvic tilt; LL, lumbar lordosis; VAS, visual analog scale; ODI, Oswestry Disability Index; SD, standard deviation; IQR, interquartile range; CI, confidence interval

Variable	Mean ± SD	Median	Mode	IQR	Min	Max	95% CI	Shapiro–Wilk p value	Skewness	Kurtosis	Shapiro–Wilk W value
Age (years)	46.2 ± 10.61	47	52	20	21	60	44.04 – 48.43	< 0.001	–0.42	–0.93	0.977
SS	36.3 ± 7.8	36.5°	36°, 38°	9.8	16.5	53	34.67 – 37.89	0.293	–0.21	–0.07	0.964
PI	51.6 ± 9.79	50.0°	44°, 47°	13.8	32	74	49.55 – 53.59	0.117	0.22	–0.31	0.972
PT	15.5 ± 6.62	16.1°	20°, 21°	10.8	2.3	30	14.14 – 16.88	0.107	–0.11	–0.87	0.985
LL	51.2 ± 10.76	51.0°	50°	12.63	19	78	49.02 – 53.47	0.378	–0.25	0.6	0.975
VAS	6.9 ± 2.2	8	9	4	2	9	6.44 – 7.36	< 0.001	–0.49	–1.16	0.913
ODI	20.5 ± 6.09	18.5	28	12	11	28	19.26 – 21.78	< 0.001	0.05	–1.66	0.896
PI–LL Mismatch	0.32 ± 8.88	–2.2°	–6.0°	12.95	–18.0	17.8	–1.51 – 2.16	0.0025	0.28	–0.86	0.967
ODI %	41.0 ± 12.17	37.00%	56%	24	22	56	38.53 – 43.56	< 0.001	0.05	–1.66	0.924

Radiographic parameters showed mean values of SS 36.3°, PI 51.6°, PT 15.5°, LL 51.2°, and a near-balanced mean PI-LL mismatch of 0.32°, although with wide variability (-18° to +17.8°). Shapiro-Wilk tests indicated that clinical outcomes such as VAS, ODI, and age were not normally distributed, whereas alignment parameters like SS, PI, PT, and LL followed approximate normality.

Correlation analysis demonstrated several significant associations. Pain (VAS) and disability (ODI) were moderately correlated (r = 0.46, p < 0.001), confirming that higher pain was accompanied by greater disability. PI-LL mismatch correlated positively with ODI (r = 0.29, p = 0.008) and weakly with VAS (r = 0.22, p = 0.06), indicating that greater alignment imbalance tended to predict worse functional outcomes. Furthermore, mismatch was strongly negatively correlated with lumbar lordosis (r = -0.78, p < 0.001) and positively with pelvic tilt (r = 0.70, p < 0.001), supporting the biomechanical principle that mismatch reflects compensatory postural mechanisms (Table 2). By contrast, PI and SS correlated strongly with structural parameters such as LL but showed little direct association with pain or disability.

**Table 2:** Correlation matrix of clinical and spinopelvic parameters VAS, visual analog scale; ODI, Oswestry Disability Index; LL, lumbar lordosis; SS, sacral slope; PT, pelvic tilt; r, pearson’s correlation coefficient; ρ, spearman’s rank correlation coefficient; p, probability value

Variables	VAS	ODI %	PI–LL Mismatch	LL	SS	PT
VAS	1	0.46 (p<0.001)	0.22 (p=0.06)	–0.10	–0.06	0.14
ODI %	1	-	0.29 (p=0.008)	–0.13	–0.11	0.19
PI–LL Mismatch	1	-	-	–0.78 (p<0.001)	0.17	0.70 (p<0.001)
LL	1	-	-	-	0.71 (p<0.001)	–0.52
SS	1	-	-	-	-	–0.62
PT	1	-	-	-	-	-

Group comparisons revealed no sex-based differences in pain, disability, or alignment parameters. Similarly, categorical analysis showed no association between sex and either LL type or high versus low disability. However, LL type (Roussouly Type 1-4) was significantly associated with structural parameters such as SS, PI, and LL (p < 0.001 for all), but not with pain (VAS) or disability scores (ODI). Thus, while the LL type describes morphological variability, it did not appear to influence clinical severity in this cohort.

Regression models identified pain intensity (VAS) and PI-LL mismatch as the most robust predictors of disability (ODI). In linear regression, each one-point increase in VAS score predicted an additional 1.44 points on the ODI (p < 0.001), while each degree increase in PI-LL mismatch added 0.25 points (p = 0.003). The model explained 38% of variance in ODI (adjusted R² = 0.336) (Table 3).

**Table 3:** Linear regression: variables predicting ODI VAS, visual analog scale; ODI, Oswestry disability index; PI–LL mismatch, pelvic incidence–lumbar lordosis mismatch; SS, sacral slope; PT, pelvic tilt; p, probability value; CI, confidence interval

Predictor	Beta (β)	95% CI	p-value	Interpretation
VAS	1.437	0.939 – 1.934	<0.001	Each 1-point ↑ in pain increases ODI by ~1.44 points
PI–LL mismatch	0.251	0.088 – 0.414	0.003	Each 1° ↑ mismatch increases ODI by 0.25 points
Age, PT, SS, Sex	NS	—	>0.1	No significant contribution

Logistic regression confirmed these findings: each one-point increase in VAS doubled the odds of severe disability (OR = 2.12, p < 0.001), while each one-degree increase in PI-LL mismatch raised the odds by 14% (OR = 1.14, p = 0.008). Age showed a borderline protective effect, with older patients slightly less likely to have high disability (OR = 0.95, p = 0.05), whereas sex remained non-significant. The logistic model demonstrated good discriminative ability (AUC = 0.817) with balanced sensitivity (72%) and specificity (70%) (Table 4).

**Table 4:** Logistic regression: predicting high disability (ODI% ≥ 40) VAS, visual analog scale; PI–LL mismatch, pelvic incidence–lumbar lordosis mismatch; OR, odds ratio; CI, confidence interval; p, probability value

Predictor	Odds Ratio (OR)	95% CI	p-value	Interpretation
VAS	2.12	1.44 – 3.13	<0.001	Each 1-point ↑ in pain doubles the odds of high disability
PI–LL Mismatch	1.14	1.03 – 1.25	0.008	Each 1° ↑ mismatch ↑ odds by 14%
Age	0.95	0.90 – 1.00	0.05	Slight protective effect
Sex	1.37	NS	0.55	Not significant

No statistically significant differences were observed between males and females for variables such as VAS, ODI, and PI-LL Mismatch (Table 5).

**Table 5:** Mann–Whitney U test results (based on gender) of VAS, ODI, and PI–LL mismatch VAS, visual analog scale; ODI, oswestry disability index; PI–LL mismatch, pelvic incidence–lumbar lordosis mismatch; U, mann–whitney u statistic; p, probability value; d, cohen’s d effect size

Variable	Test Used	p-value	U value	Effect Size (Cohen’s d)	Interpretation
VAS	Mann–Whitney U	0.222	10.5	~0.28 (small)	Not significant
ODI	Mann–Whitney U	0.858	13	Negligible	Not significant
PI–LL mismatch	Mann–Whitney U	0.351	7	Negligible	Not significant

Taken together, these results highlight that while spinopelvic morphology varies across individuals, the clinically meaningful markers of disability in CNSLBP are pain severity and PI-LL mismatch. Morphological LL, such as Roussouly LL Types 1-4, and static parameters like PI, describe structural anatomy but do not translate into clinical severity. Instead, PI-LL mismatch reflects biomechanical inefficiency and emerges as a quantifiable risk factor for functional impairment. In clinical terms, routine evaluation of PI-LL mismatch alongside pain scoring may provide valuable insight into patients at higher risk of disability, supporting more targeted and biomechanically oriented management strategies.

## Discussion

CNSLBP is a complex condition, and sagittal spinopelvic alignment is one of the key factors linked to its symptoms and functional limitations. Among the alignment parameters, the relationship between PI and LL (PI-LL mismatch) has been shown to influence both pain and functional limitation. When the lumbar curve does not match the PI, abnormal stress is placed on spinal and paraspinal structures, leading to discomfort and disability. In our study, PI-LL mismatch showed a consistent association with higher pain and disability scores, supporting earlier research that sagittal malalignment correlates with symptom severity [15-17].

PI is a fixed anatomical determinant of sagittal alignment [18]. When LL does not match PI, a biomechanical imbalance occurs, producing shear forces, abnormal loading, and compensatory muscle activation, which over time may result in pain and degeneration [8-10]. Traditionally, a PI-LL mismatch of more than 10° has been considered clinically relevant [19-21]. However, our results suggest that even smaller mismatches can be clinically meaningful in CNSLBP, where disability was observed despite mean mismatch values close to neutral. This highlights the need to consider mismatch relative to individual pelvic morphology rather than relying only on fixed cutoffs or the threshold value of 10°.

Our results are in line with the findings of Bourret et al. (2022), who studied a large group of asymptomatic individuals and showed that the PI-LL mismatch is not fixed, but according to the PI; they proposed that the ideal mismatch changes according to each person’s PI [22]. Similar to their observation, our study also indicates that alignment must be viewed in an individualized manner. This opens new avenues for future research, where treatment targets could be based on patient-specific PI rather than applying the same standard to all.

The role of age has also been debated in sagittal alignment studies. Niu et al. (2024) examined a larger population with CNSLBP and found that increasing age was strongly related to sagittal imbalance and disability [17]. In contrast, our study did not show a significant correlation between age and disability. One reason for this difference may be that, in our study, the age profile was 21-60 years, who were mostly in the younger and middle-aged range. Niu et al.’s cohort included a wider age spectrum of 18-84 years with more degenerative changes, where age-related imbalance becomes more prominent. This suggests that PI-LL mismatch and pain may be more important predictors of disability in younger patients, while age plays a greater role in older degenerative spines.

This study has some limitations. Its cross-sectional design prevents us from establishing cause and effect. The sample size was moderate and limited to a single center, so the findings may not apply to all populations. We also did not include other sagittal parameters, such as sagittal vertical axis or thoracic kyphosis, which are known to influence overall balance. Although our findings support a relationship between sagittal imbalance with pain severity and functional outcome, some prior studies have reported weak or inconsistent correlations [17,23,24]. These inconsistencies may stem from differences in sample demographics, chronicity of symptoms and disability, or radiographic methods used for spinopelvic evaluation. Despite these limitations, our results add to the growing evidence that PI-LL mismatch should not be treated as a single fixed number but should be understood in accordance with each patient’s PI.

In summary, our study supports the idea that PI-LL mismatch is an important marker of pain and disability in CNSLBP. Instead of applying a universal threshold, future research should explore personalized alignment targets that respect individual PI. Longitudinal studies and interventional trials may help determine whether restoring patient-specific balance (PI-LL mismatch) can improve outcomes, both in conservative and surgical management.

## Conclusions

Our study demonstrates that PI-LL mismatch has a significant role in the severity of pain and disability in patients with CNSLBP. Our results show that even smaller mismatches can be clinically meaningful and should be interpreted in accordance with an individual's PI. We also observed that age was not strongly related to disability in our cohort, which may reflect the younger patient population. These findings support the idea that sagittal balance should be individualized rather than judged against universal thresholds. This approach opens the possibility for future research to explore patient-specific alignment targets and to determine whether interventions directed at restoring personalized balance can improve long-term outcomes.
